# Cytokine Responses to the Anti-schistosome Vaccine Candidate Antigen Glutathione-S-transferase Vary with Host Age and Are Boosted by Praziquantel Treatment

**DOI:** 10.1371/journal.pntd.0002846

**Published:** 2014-05-08

**Authors:** Claire D. Bourke, Norman Nausch, Nadine Rujeni, Laura J. Appleby, François Trottein, Nicholas Midzi, Takafira Mduluza, Francisca Mutapi

**Affiliations:** 1 Institute of Immunology & Infection Research, Centre for Immunity, Infection & Evolution, School of Biological Sciences, University of Edinburgh, Edinburgh, United Kingdom; 2 Centre d'Infection et d'Immunité de Lille, Inserm U 1019, Institut Pasteur de Lille, Université Lille Nord de France, Lille, France; 3 National Institute of Health Research, Harare, Zimbabwe; 4 Biochemistry Department, University of Zimbabwe, Harare, Zimbabwe; Centers for Disease Control and Prevention, United States of America

## Abstract

**Background:**

Improved helminth control is required to alleviate the global burden of schistosomiasis and schistosome-associated pathologies. Current control efforts rely on the anti-helminthic drug praziquantel (PZQ), which enhances immune responses to crude schistosome antigens but does not prevent re-infection. An anti-schistosome vaccine based on *Schistosoma haematobium* glutathione-S-transferase (GST) is currently in Phase III clinical trials, but little is known about the immune responses directed against this antigen in humans naturally exposed to schistosomes or how these responses change following PZQ treatment.

**Methodology:**

Blood samples from inhabitants of a *Schistosoma haematobium*-endemic area were incubated for 48 hours with or without GST before (n = 195) and six weeks after PZQ treatment (n = 107). Concentrations of cytokines associated with innate inflammatory (TNFα, IL-6, IL-8), type 1 (Th1; IFNγ, IL-2, IL-12p70), type 2 (IL-4, IL-5, IL-13), type 17 (IL-17A, IL-21, IL-23p19) and regulatory (IL-10) responses were quantified in culture supernatants via enzyme-linked immunosorbent assay (ELISA). Factor analysis and multidimensional scaling were used to analyse multiple cytokines simultaneously.

**Principal Findings:**

A combination of GST-specific type 2 (IL-5 and IL-13) and regulatory (IL-10) cytokines was significantly lower in 10–12 year olds, the age group at which *S. haematobium* infection intensity and prevalence peak, than in 4–9 or 13+ year olds. Following PZQ treatment there was an increase in the number of participants producing detectable levels of GST-specific cytokines (TNFα, IL-6, IL-8, IFNγ, IL-12p70, IL-13 and IL-23p19) and also a shift in the GST-specific cytokine response towards a more pro-inflammatory phenotype than that observed before treatment. Participant age and pre-treatment infection status significantly influenced post-treatment cytokine profiles.

**Conclusions/Significance:**

In areas where schistosomiasis is endemic host age, schistosome infection status and PZQ treatment affect the cellular cytokine response to GST. Thus the efficacy of a GST-based vaccine may also be shaped by the demographic and epidemiological characteristics of targeted populations.

## Author Summary

Schistosomiasis is caused by infection with *Schistosoma* spp. parasites, for which the main treatment is the drug praziquantel (PZQ). Since PZQ does not prevent reinfection, an anti-schistosome vaccine based on the *Schistosoma haematobium* enzyme glutathione-S-transferase (GST) is being developed. In this study we investigated the GST-specific immune responses of people naturally exposed to schistosomes and the affect that PZQ has on these responses. We cultured blood samples from a schistosome-exposed community with GST before and six weeks after PZQ treatment and measured a range of soluble proteins (cytokines) in culture supernatants as indicators of blood cell activation and phenotype. Before treatment, GST-specific cytokine responses varied with host age, particularly in children with high intensity schistosome infections. Following treatment, GST activated blood samples from more individuals to produce a broader range of cytokines and the combination of GST-specific cytokine responses reflected a more pro-inflammatory immune phenotype than that observed pre-treatment. Post-treatment responses varied according to host age and pre-treatment infection status. Taken together, our study suggests that current and future GST-based vaccine trials should take host age, schistosome infection status and PZQ treatment history into account since these factors influence GST-specific immune activation.

## Introduction

Over 200 million people in 74 countries are currently infected with *Schistosoma* spp. parasites, which are responsible for an estimated 15, 000 deaths and 1.76 million disability adjusted life years per annum [1]–[3]. *Schistosoma haematobium* is the causative agent of urogenital schistosomiasis which results from pathological immune responses to eggs excreted into the bladder and genital tract of their host by adult parasites residing in the adjacent venules. Effective schistosome control is required to alleviate schistosome-associated pathologies, to protect the 650 million people currently at risk from schistosome infection and to reach the estimated 88% of infected people currently without access to drug treatment [2], [4]. Current control efforts rely on treatment with the anti-helminthic drug praziquantel (PZQ), which has reported cure rates of over 80% [5], [6] and can reduce the risk of urogenital lesions if administered during childhood [7]. There is also mounting evidence that PZQ boosts both innate and adaptive immune responses to schistosome antigens [8]–[10] due to increased worm death in the bloodstream and an associated increase in exposure of schistosome antigens to immune recognition after treatment [11], [12]. However, although there is some evidence that this immunological boost promotes a degree of resistance to re-infection in humans [10], [13], both infection prevalence and associated pathologies return after treatment and therefore repeated treatment is required [14], [15]. For nearly 30 years an anti-schistosome vaccine has been seen as a desirable long-term adjunct to drug treatment [3], [16]. More recently, it has been proposed that a combination of PZQ treatment and an anti-pathology vaccine may improve schistosome control [12], [16], [17].

The 28 KDa *S. haematobium* vaccine candidate antigen glutathione-S-transferase (GST [18], [19]) is a multifunctional enzyme expressed on the tegument and sub-tegument of adult worms [20] and larval schistosomes [21] and the current focus of vaccine trials in humans. The exact function of schistosome GST is unknown but its role in fatty acid metabolism and prostaglandin D_2_ synthesis may contribute to immune evasion by the parasite [19]. GST-based vaccination has been extensively studied in animal models, leading to a reduction in parasite fecundity in cattle [22], goats [23] and primates [24], which has been attributed to production of antibodies that neutralise GST enzyme activity [25]–[27]. Importantly, reducing egg production by adult schistosomes is an effective means of reducing immunopathology since schistosomes do not replicate in their definitive hosts [3]. The latter is supported by observations in *S. haematobium* infected Patas monkeys where bladder lesion intensity was reduced following GST vaccination [24], [28]. A recent Phase I randomised clinical study has shown that elevated levels of GST-neutralising antibodies, which are associated with reduced parasite fecundity [25], [29], as well as increased peripheral blood cytokine responses were detectable 21 days after a double dose of the GST vaccine was administered to healthy Caucasian adult males [30]. Furthermore, GST-specific PBMC cytokine responses in the latter study appeared to be biased towards a CD4+ T helper (h) 2 phenotype [30], which is associated with protective immune responses to schistosome homogenate antigens in cohorts endemically-exposed to *S. haematobium* [31], [32]. Despite these promising observations in animal models and safety-immunogenicity trials in humans in a non-endemic setting, very little is known about the cellular immune phenotype elicited by purified GST in naturally schistosome-exposed populations who would be the target recipients of a GST-based vaccine. In particular, GST-specific cytokine responses have been investigated in *S. haematobium*-exposed adults [33], but no studies to date have investigated the age-distribution of GST-specific cellular cytokine responses which is closely related to schistosome exposure history [11], [34]. Furthermore, despite speculation that GST-based vaccine efficacy may be enhanced by co-administration with PZQ in human populations [12], [16], [17], [35] there is no existing data on how PZQ treatment affects GST-specific cytokine responses.

The aim of this study was to address two previously un-addressed hypotheses regarding cellular cytokine responses to purified GST in an *S. haematobium*-endemic community: firstly that these responses vary with age (and by proxy, exposure to infection [34]), and secondly that they are boosted by PZQ treatment. Importantly, we have made use of statistical approaches that integrate data on multiple cross-regulatory cytokines associated with relevant cellular immune phenotypes (innate inflammatory, Th1, Th2, Th17 and regulatory responses) so that our analysis considers not only the dynamics of individual GST-specific cytokines, but also their patterns of production relative to one another.

## Methods

### Ethical permissions

Ethical approval was granted by the Medical Research Council of Zimbabwe and the University of Zimbabwe's Institutional Review Board. Local permission for the study was granted by the Provincial Medical Director of Mashonaland East. All prospective participants were informed of the study aims and procedures in their local language (Shona). All adult participants provided informed written consent and children were recruited only if informed written consent was provided by a parent or guardian.

### Study design

The current study is part of on-going schistosome immuno-epidemiological research in Murehwa District, Mashonaland East province, Zimbabwe where *S. haematobium* is endemic [36]–[38]. Pre-studies in the area showed that *S. mansoni* and soil-transmitted helminth (STH) prevalence is low (<2%) and the region is classified as a low transmission area for *Plasmodium* spp. [39]. The study area had not been included in previous PZQ treatment programs. Baseline recruitment of participants was school-based but pre-school age children and adults were also informed of the study and invited to attend via community meetings prior to the commencement of baseline sampling.

The following samples were collected from all recruited participants (n = 284); 1) a minimum of 2 urine samples collected over 3 consecutive days for quantification of *S. haematobium* infection intensity, 2) a minimum of 2 stool samples collected over 3 consecutive days for quantification of soil-transmitted helminth (STH) and *S. mansoni* infections, and 3) 10 ml venous blood. Participants also completed a questionnaire to assess residential history, patterns of exposure to schistosome infective water and anti-helminthic drug treatment history. Participants were excluded from the study if: they did not provide samples 1–3 (n = 5), they provided insufficient blood volume for stimulation with GST and a control culture without antigen (n = 13) or quantification of all cytokines (n = 25), they indicated in their questionnaire responses that they were not life-long permanent residents of the study area (n = 23), or they were positive for STH (n = 0), *S. mansoni* (n = 5), HIV (n = 18) or *Plasmodium* spp. infection (n = 0).

After baseline sampling all compliant participants were treated with a single dose of PZQ (40 mg/kg body weight) and sampling was repeated 6 weeks post-treatment. For inclusion in the post-treatment cohort participants were required to provide a full set of samples 1–3 and remain negative for all co-infections 6 weeks post-treatment (n = 126). Ten eligible participants refused PZQ treatment for religious reasons and 9 eligible participants remained positive for *S. haematobium* infection following treatment, these participants were excluded from the post-treatment cohort. Based on these criteria, a total of 195 participants were included in the baseline cohort and, of these, 107 participants made up the post-treatment cohort. Re-infection was assessed at 6 and 18 months post-treatment in participants who provided samples 1 and 2 at these timepoints (n = 75). High community-wide prevalence and infection intensity at baseline precluded inclusion of an untreated control group according to WHO treatment guidelines [40].

### Diagnostic tests

Stool and urine samples were collected and processed following the standard microscopic procedures (Kato Katz for stool and urine filtration methods for urine) [41], [42]. Infection intensity was expressed as the mean egg count per 10 ml urine for *S. haematobium* calculated from a minimum of 2 samples/participant before treatment, and 6 weeks, 6 months and 18 months post-treatment. *Plasmodium* spp. and HIV infection were identified serologically as previously described [10].

### Schistosome antigens

Recombinant 28 KDa GST of a Senegalese strain of *S. haematobium* was cloned and purified using previously described protocols [43]. GST preparations were confirmed to be endotoxin free (<0.015 EU/ml) using the Limulus amebocyte lysate assay (Sigma-Aldrich, Lyon, France).

### Whole blood culture

Venous blood samples were collected from study participants into EDTA-coated tubes and cultured at a 1∶3 dilution with media (RPMI 1640 supplemented with 2 mM L-glutamine and 100 U Penicillin/Streptomycin (all Lonza, Verviers, Belgium)) in duplicate wells coated with either 2 µg/ml GST or without antigen (i.e. culture media alone) for 48 hours at 37°C in Anaerogen Compact anaerobic atmosphere generation pouches (OXOID, Basingstoke, U.K.). Cell-free culture supernatants were frozen and assayed within 12 months.

### Cytokine ELISA

Interferon (IFN) γ, Tumour necrosis factor (TNF) α, Interleukin(IL)-2, IL-4, IL-5, IL-6, IL-8, IL-10, IL-12p70, IL-13 and IL-21 (BD Biosciences, Oxford, U.K.), IL-17A and IL23p19 (eBiosciences Ltd., Hatfield, U.K.) were assayed in culture supernatants via enzyme-linked immunosorbent assay (ELISA) using published protocols [10].

### Statistical analysis

*S. haematobium* infection intensity exhibited a negatively skewed distribution within the study cohort as is typical of schistosome infection in endemic populations [44]. Infection intensity was therefore log10(x+1) transformed and compared for infected participants by gender (male and female) and age group (4–9, 10–12 and 13+ years) via ANOVA (adjusted sums of squares). Post-hoc pairwise comparisons between the 3 age groups were made using Fisher's least significant difference test.

Cytokine levels present in culture supernatants were not normally distributed even following transformation, therefore all comparisons of cytokine levels were conducted using non-parametric statistical tests. Cytokine levels in GST-stimulated cultures were compared to those present in parallel cultures without antigen from the same individuals via the paired Wilcoxon test. Cytokine levels present in un-stimulated culture supernatants were then subtracted from those present in GST-stimulated cultures to give GST-specific cytokine levels. Where an individual did not produce a cytokine at levels above those in their parallel un-stimulated culture they were assigned a value of 0 pg/ml for that cytokine. The percentage of GST-specific cytokine producers (i.e. levels above those in un-stimulated cultures) was compared by gender, age group and *S. haematobium* infection status via Pearson's Chi-squared test. Comparisons between the percentage of GST-specific cytokine producers pre- and 6 weeks post-treatment were made using the paired McNemar's test. To confirm that treatment-related differences in cytokine responses were not due to sampling bias in the smaller post-treatment cohort, baseline GST-specific cytokine levels were compared between participants included in post-treatment follow up analyses and those that were not included using the Mann Whitney U test (p>0.05 for all 13 cytokine responses; data not shown).

GST-specific cytokine responses were reduced into a smaller number of variables (principal components, PCs) according to their shared patterns of expression via factor analysis as previously reported [10]. Due to the skewed nature of GST-specific cytokine responses (i.e. few producers for some cytokines and a non-normal distribution of cytokine levels) values were log10(x+1) transformed to minimise the influence of outlier values [45] and only GST-specific cytokines that were detectable in >30% of participants were included in the factor analyses. Dynamics of cytokines with a factor loading ≥0.5 or ≤−0.5 onto a PC were considered to be reflected by that PC. Separate factor analyses were conducted for baseline (Table S1) and 6 weeks post-treatment cytokine responses (Table S2). PC regression factor scores at each timepoint were compared by gender, age group and *S. haematobium* infection status via ANOVA. Sequential sums of squares were used to account for demographic variation (gender and age group) before infection status.

To characterise changes in the distribution of GST-specific cytokine responses for each participant 6 weeks after treatment relative to pre-treatment levels non-metric multidimensional scaling (NMS) was used to provide a visual representation of similarity/dissimilarity between participant responses. NMS was conducted as described previously [10], [46] and the non-parametric multiple response permutation procedure (MRPP) was used to statistically compare pre- and post-treatment cytokine profiles. Pearson's correlation analysis was used to determine the amount of variation between participant NMS scores that were attributable to each spatial axis. Non-parametric Kendall correlations analysis was used to identify associations between NMS spatial axes and the original concentrations of the 13 individual cytokines. A cytokine was considered to be reflected by the spatial axis if Kendall's tau (τ) ≥0.4.

Statistical tests were conducted using SPSS Statistics version 19 software (IBM, Hampshire, U.K.) and NMS was implemented using PC-ORD software (MJM Software Design, Gleneden Beach, U.S.A.) [46]. Comparisons were considered to be significant at p<0.05. Where >10 comparisons were made the p-value was adjusted for multiple comparisons via the sequential Bonferroni correction and comparisons that were significant post-correction were considered highly significant.

## Results

### *S. haematobium* infection distribution in the study cohort

**Figure 1:**
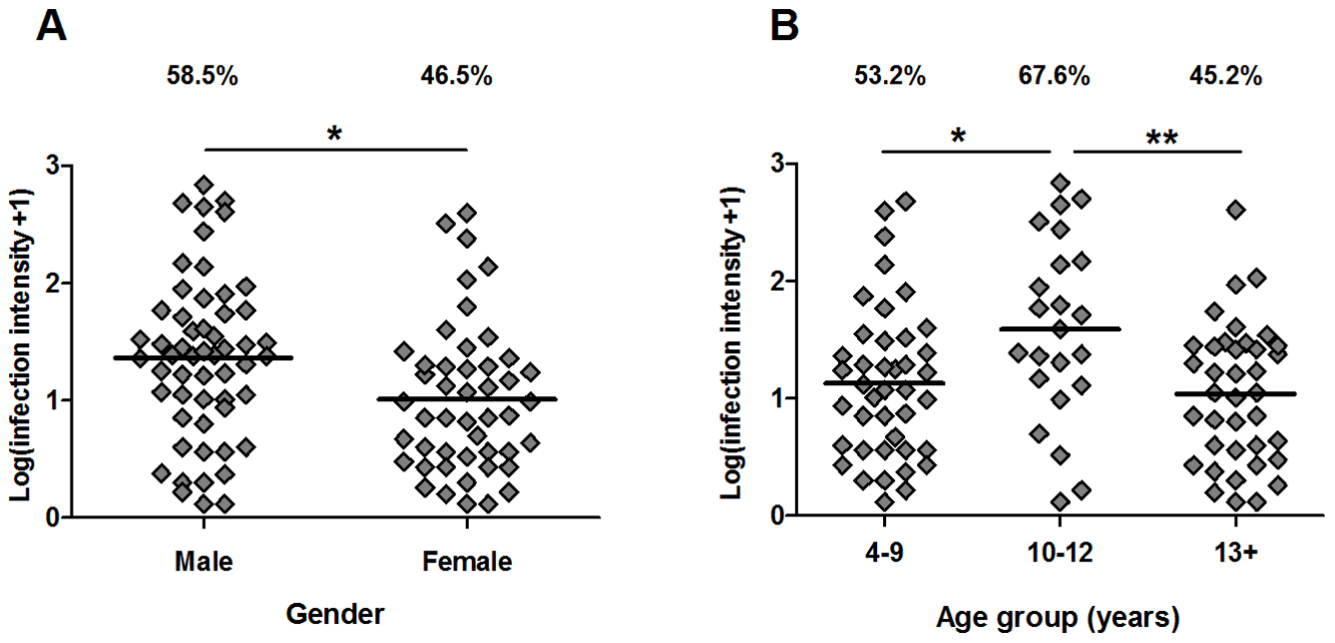
Distribution of *S. haematobium* infection in the study cohort. Infection prevalence (%) and intensity (Log10(x+1) transformed mean egg counts/10 ml urine) sub-divided by gender (**A**) and age group (**B**). Mean values are indicated by horizontal bars. Significance levels from ANOVA (adjusted sums of squares) of infection intensity for infected participants are shown for gender and post-hoc pair-wise Fisher's least significant difference comparisons are shown for the 3 age groups (n = 101);*p<0.005, **p<0.01.

The baseline study cohort consisted of 94 males and 101 females ranging in age from 4–84 years. Baseline *S. haematobium* infection prevalence was 52.3%. A higher percentage of men (58.5%) than women (46.5%) were *S. haematobium* positive at baseline, and of the infected individuals men had a significantly higher mean infection intensity than women (Figure 1A; F_1, 96_: 6.56, p = 0.012). *S. haematobium* infection intensity also showed a non-linear relationship with age (Figure 1B: F_2, 96_: 4.00, p = 0.021) as is typical of schistosome epidemiology [44], with infection intensity peaking in children aged 10–12 years (4–9 vs. 10–12 years: Mean difference: −0.385, p = 0.028; 13+ vs. 10–12 years: Mean difference: −0.486, p = 0.007). The demographic and *S. haematobium* infection characteristics of the study cohort are summarised in Table 1.

**Table 1:** Demographic and *S. haematobium* infection characteristics of the study cohort by age group.

		Age group (years)		
		4–9	10–12	13+
**n**		77	34	84
**Gender**	Males, Females	38, 39	18, 16	38, 46
**Age**	Mean ± SEM	7.25±0.15	11.35±0.12	26.92±2.11
***S. haematobium*** **infection intensity**	Mean egg counts ± SEM (min.-max.)	24.04±8.70 (0–481)	86.85±28.61 (0–692)	12.87±5.10 (0–403)
***S. haematobium*** **prevalence**	Prevalence (%)	53.2	67.6	45.2
**GST-specific cytokines**	Producers^a^ (%)	97.4	94.1	94.0

a Detectable GST-specific cytokine production>levels present in un-stimulated cultures for ≥1 of the 13 cytokines assayed.

### GST-specific cytokine responses vary with age

All cytokines were present at significantly higher levels in GST-stimulated whole blood cultures than in corresponding un-stimulated cultures (p<0.001 for Wilcoxon comparisons of all 13 cytokines; data not shown) indicating that a specific cytokine response to *S. haematobium* GST was elicited within the cohort. Only 4.6% of participants (n = 9) produced no detectable GST-specific cytokines at levels greater than those present in un-stimulated cultures (Table 1).

Having established that GST elicited a whole blood cytokine response, we next sought to characterise demographic factors that may influence these responses. We first compared the percentage of participants producing GST-specific cytokines and found no difference according to gender or infection status in any of the cytokines measured (Table 2). However, when compared by age, the youngest age group (4–9 years) was found to have a significantly higher percentage of GST-specific IL-4 (*X^2^*: 14.08, p = 0.001) and IL-10 (*X^2^*: 8.49, p = 0.014) producers than either the 10–12 or 13+ year age groups (Figure S1, Table 2).

**Table 2:** Pearson's Chi-squared comparison of the percentage of participants producing detectable levels of GST-specific cytokines by gender, age group and *S. haematobium* infection status before anti-helminthic treatment.

		Gender		*X^2^ (p)*	Age group (years)			*X^2^ (p)*	Infection status		*X^2^ (p)*
	Cytokine	Male (n = 94)	Female (n = 101)	df:1	4–9 (n = 77)	10–12 (n = 34)	13+ (n = 84)	df:2	Un-infected (n = 93)	Infected (n = 102)	df:1
**Innate Inflammatory**	**TNFα**	39.4	32.7	0.947 (0.331)	44.2	26.5	32.1	4.110 (0.128)	31.2	40.2	1.717 (0.190)
	**IL-6**	74.5	64.4	2.337 (0.126)	74.0	76.5	61.9	3.784 (0.151)	74.2	64.7	2.056 (0.152)
	**IL-8**	43.6	35.6	1.295 (0.255)	39.0	52.9	34.5	3.451 (0.178)	43.0	36.3	0.924 (0.336)
**Th1**	**IFNγ**	35.1	35.6	0.006 (0.938)	36.4	38.2	33.3	0.308 (0.857)	36.6	34.3	0.107 (0.743)
	**IL-2**	28.7	24.8	0.393 (0.531)	32.5	20.6	23.8	2.318 (0.134)	25.8	27.5	0.067 (0.795)
	**IL-12p70**	39.4	29.7	2.014 (0.156)	37.7	44.1	27.4	3.622 (0.164)	37.6	31.4	0.846 (0.358)
**Th2**	**IL-4**	19.1	19.8	0.013 (0.908)	32.5	14.7	9.5	**14.079 (0.001** ** **)**	17.2	21.6	0.591 (0.442)
	**IL-5**	39.4	40.6	0.031 (0.861)	44.2	26.5	41.7	3.244 (0.197)	40.9	39.2	0.055 (0.815)
	**IL-13**	31.9	31.7	0.001 (0.972)	35.1	17.6	34.5	3.806 (0.149)	33.3	30.4	0.194 (0.660)
**Th17**	**IL-17A**	19.1	19.8	0.13 (0.908)	22.1	20.6	16.7	0.782 (0.677)	21.5	17.6	0.462 (0.497)
	**IL-21**	51.1	44.6	0.827 (0.363)	51.9	38.2	47.6	1.778 (0.411)	48.4	47.1	0.034 (0.853)
	**IL-23**	58.5	55.4	0.187 (0.666)	64.9	47.1	53.6	3.750 (0.153)	62.4	52.0	2.148 (0.143)
**Regulatory**	**IL-10**	38.3	37.6	0.009 (0.923)	46.8	17.6	38.1	**8.487 (0.014** * **)**	36.6	39.2	0.146 (0.703)

Cytokines are categorised according to the cellular phenotype with which they are most commonly associated. Pearson's Chi-squared analysis: p-values<0.05 are indicated in bold and those significant after Bonferroni correction for multiple comparison are indicated by underlined text (correction conducted separately for each factor).

*p<0.05,

**p<0.01.

**Figure 2:**
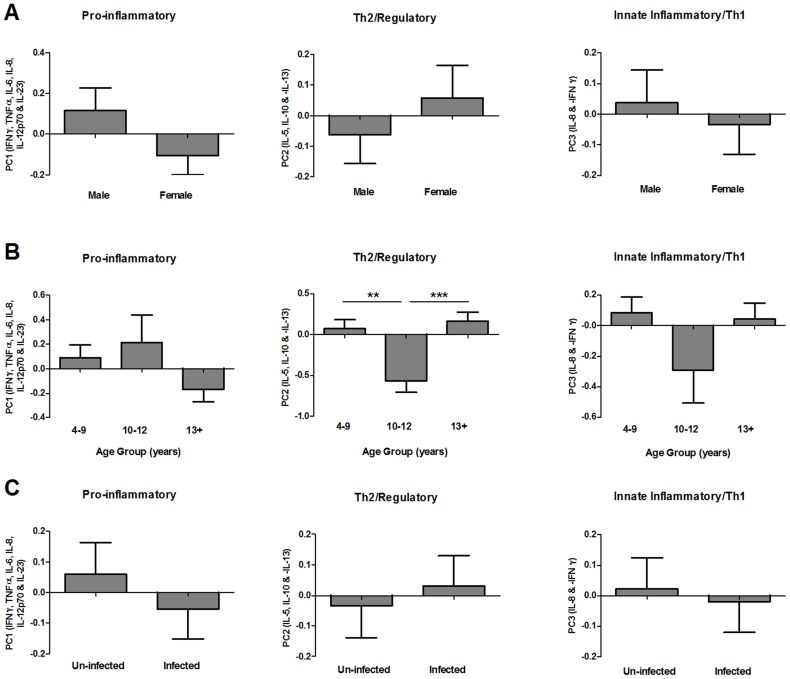
Distribution of pre-treatment GST-specific cytokine profiles by gender, age group and *S. haematobium* infection status. Mean principal component regression factor scores for pro-inflammatory (PC1), Th2/regulatory (PC2) and innate inflammatory/Th1 (PC3) cytokine profiles sub-divided by gender (**A**), age group (**B**) and schistosome infection status (**C**; un-infected = egg negative in all urine samples, infected = egg positive in one or more urine samples). Where ANOVA (sequential sums of squares accounting for variation in gender and age group before infection status) indicated that a factor significantly contributed to variation in cytokine profiles, pair-wise comparisons of PC scores were conducted via post-hoc Fisher's least significant difference tests between the 3 age groups (n = 195);**p<0.01, ***p<0.001. Error bars: standard error of the mean.

In addition to the presence/absence of individual cytokines, the relative levels of different cross-regulatory cytokines could also be an important determinant of the GST-specific immune response. To characterise patterns of GST-specific cytokines, all cytokines produced by >30% of participants (i.e. IFNγ, TNFα, IL-5, IL-6, IL-8, IL-10, IL-12p70, IL-13, IL-21 and IL23p19), were reduced into PCs according to their shared patterns of expression via factor analysis. This analysis identified 3 key patterns of GST-specific cytokine response accounting for variation between participants; PC1 accounted for the largest amount of variation (28.5%) and corresponded to pro-inflammatory cytokine responses (IFNγ, TNFα, IL-6, IL-8, IL-12p70 and IL23p19), PC2 corresponded to a combination of Th2 (IL-5 and IL-13) and regulatory (IL-10) responses and PC3 reflected expression of the neutrophil chemoattractant (IL-8) and was negatively associated with the Th1 cytokine IFNγ (for factor loadings refer to Table S1). None of these cytokine patterns varied according to participant gender or infection status (Figure 2; Table 3), however PC2 significantly differed between the 3 age groups (Figure 2; F_2, 188_: 6.940, p = 0.001). Pair-wise comparisons between age groups indicated that PC2 scores were significantly lower in 10–12 year olds than in either 4–9 (Mean difference: −0.678, p = 0.002) or 13+ year olds (Mean difference: −0.777, p<0.001) indicating a lower Th2/regulatory cytokine response to GST in this group. PCs 1 and 3 did not significantly differ between the 3 age groups (Figure 2; Table 3).

**Table 3:** Comparison of pre- and post-treatment GST-specific cytokine profiles at baseline by gender, age group and baseline *S. haematobium* infection status.

		Gender				Age group (years)						Baseline infection status		
Pre-treatment		Male (n = 94)	Female (n = 101)	F_1, 188_	p	4–9 (n = 77)	10–12 (n = 34)	13+ (n = 84)	F_2, 188_	p	Un-infected (n = 93)	Infected (n = 102)	F_1, 188_	p
**PC1**	**Pro-inflammatory**	0.115	−0.107	2.444	0.120	0.091	0.212	−0.169	2.166	0.117	0.059	−0.054	1.692	0.195
**PC2**	**Th2/Regulatory**	−0.063	0.058	0.753	0.387	0.071	−0.564	0.164	6.940	**0.001** **	−0.034	0.031	1.286	0.258
**PC3**	**Innate Inflammatory/Th1**	0.038	−0.035	0.258	0.612	0.082	−0.293	0.043	1.838	0.162	0.022	−0.020	0.011	0.915

Post-treatment		Male (n = 54)	Female (n = 53)	F_1, 102_	p	4–9 (n = 59)	10–12 (n = 20)	13+ (n = 28)	F_2, 102_	p	Un-infected (n = 48)	Infected (n = 59)	F_1, 102_	p
**PC1**	**Pro-inflammatory**	−0.989	0.103	1.119	0.293	0.223	−0.269	−0.287	3.547	**0.032** *	0.017	−0.014	0.003	0.954
**PC2**	**Th2/Th17/Regulatory**	−0.103	0.107	1.240	0.268	0.102	−0.319	0.002	1.501	0.228	0.285	−0.227	6.070	**0.015** *
**PC3**	**Th2/Regulatory**	0.117	−0.121	1.479	0.227	0.075	−0.052	−0.122	0.385	0.682	−0.083	0.066	0.404	0.526

Cytokines are categorised according to the cellular phenotype with which they are most commonly associated. Mean pre- and post-treatment PC regression scores are indicated for each group. ANOVA (sequential sums of squares) analysis accounted for variation in PC scores due to gender and age group before baseline infection status,

*p<0.05,

**p<0.01.

### Anti-helminthic treatment boosts cytokine production in response to GST

Laboratory studies have proposed that co-administration of a GST-based vaccine with PZQ may promote vaccine efficacy [12], [16], [17], therefore we investigated whether naturally-acquired GST-specific cytokine responses are affected by PZQ treatment. Six weeks after a single dose of PZQ there was a significant increase relative to baseline in the percentage of participants producing detectable levels of GST-specific cytokines associated with innate inflammatory (TNFα, IL-6 and IL-8), Th1 (IFNγ, IL-2 and IL-12p70), Th2 (IL-13) and Th17 (IL-23p19) immune responses (Figure S2; Table 4).

**Table 4:** Paired comparison of the percentage of participants producing detectable levels of GST-specific cytokines before and 6 weeks after PZQ treatment.

		%Cytokine Producers		McNemar's test (p)
		Pre-treatment (n = 107)	Post-treatment (n = 107)	df:1
**Innate Inflammatory**	**TNFα**	40.6	76.4	**≤0.001** ***
	**IL-6**	76.4	92.5	**0.002** **
	**IL-8**	44.3	91.5	**≤0.001** ***
**Th1**	**IFNγ**	35.8	71.7	**≤0.001** ***
	**IL-2**	27.4	47.2	**0.005** **
	**IL-12p70**	32.1	71.7	**≤0.001** ***
**Th2**	**IL-4**	20.8	23.6	0.749
	**IL-5**	38.7	43.4	0.568
	**IL-13**	32.1	61.3	**≤0.001** ***
**Th17**	**IL-17A**	18.9	30.2	0.096
	**IL-21**	43.4	55.7	0.124
	**IL-23**	61.3	93.4	**≤0.001** ***
**Regulatory**	**IL-10**	43.4	42.5	1

Cytokines are categorised according to the cellular phenotype with which they are most commonly associated. McNemar's test (paired data, binomial distribution): p-values<0.05 are indicated in bold and those significant after Bonferroni correction for multiple comparison are indicated by underlined text,

**p<0.01,

***p<0.001.

**Figure 3:**
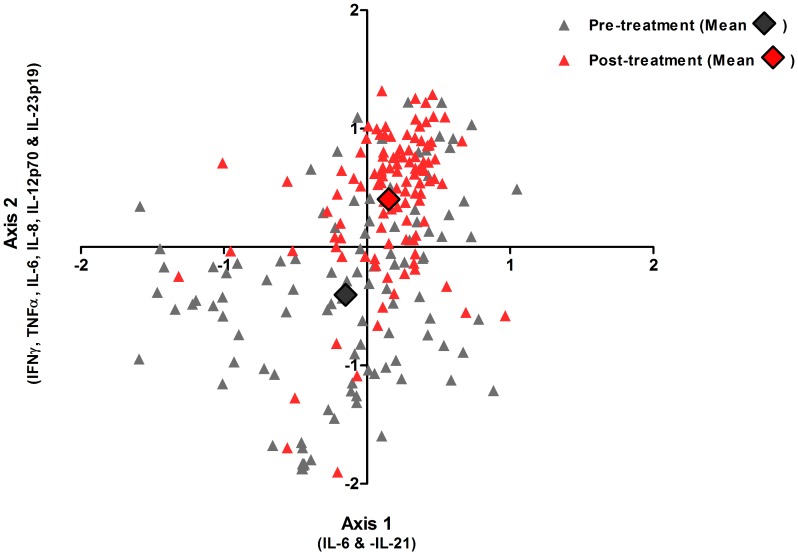
Relative similarity and dissimilarity in patterns of GST-specific cytokine responses before and 6 weeks after praziquantel treatment. NMS ordination plot showing Sorensen Bray-Curtis distance scores (an indicator of how a participant's combination of 13 GST-specific cytokine responses rank relative to those of all other participants) for all participants plotted before (**grey triangles**) and 6 weeks after (**red triangles**) praziquantel treatment. Axis 1 and 2 are spatial meaning that participants positioned close together have similar patterns of cytokine responses to GST and those positioned far apart have dissimilar patterns of cytokine responses to GST. The individual cytokines that most closely correlate with each axis are indicated. Bold diamonds represent the mean pre- (**grey**) and post-treatment (**red**) scores for the study cohort (n = 107).

In addition to an increase in the proportion of individuals producing cytokines in response to GST, we also investigated whether there was a post-treatment shift in combined cytokine responses to GST relative to pre-treatment patterns. The latter is an important addition to our understanding of GST-specific immune responses since post-treatment cytokine phenotype appears to be a determinant of resistance to re-infection both in human population studies [10], [47] and murine models of schistosomiasis [48]. To visualise this comparison NMS was used to position each participant along two spatial axes according to their levels of all 13 GST-specific cytokines relative to those of all other participants both before and 6 weeks after treatment. Thus participants with similar combinations of GST-specific cytokines are arranged close together and those with dissimilar responses are further apart. The ordination plots of this analysis showed that pre- and post-treatment cytokine responses formed distinct clusters reflecting a shift in the whole blood cytokine responses elicited by GST (Figure 3) and this dissimilarity between pre- and post-treatment cytokine responses was statistically significant (MRPP; T: −53.438, p<0.001, A: 0.062). Kendall's correlation between the original cytokine levels and the NMS spatial axes indicated that the majority of variation in pre- and post-treatment cytokine responses was attributable to the increase in levels of IFNγ (τ: 0.449), TNFα (τ: 0.508), IL-6 (τ: 0.782), IL-8 (τ: 0.544), IL-12p70 (τ: 0.500) and IL23p19 (τ: 0.617) 6 weeks after treatment (Axis 2; Pearson's r^2^: 0.492). To a lesser extent pre- and post-treatment cytokine responses also varied along Axis 1 (Pearson's r^2^: 0.236), which was positively correlated with IL-6 (τ: 0.440) and negatively correlated with IL-21 (τ: −0.391). These cytokines are associated with innate inflammatory and effector Th1 and Th17 responses and thus the phenotype of the post-treatment cytokine response to GST is more pro-inflammatory than at baseline.

### Post-treatment cytokine responses vary according to host age and pre-treatment infection status

**Figure 4:**
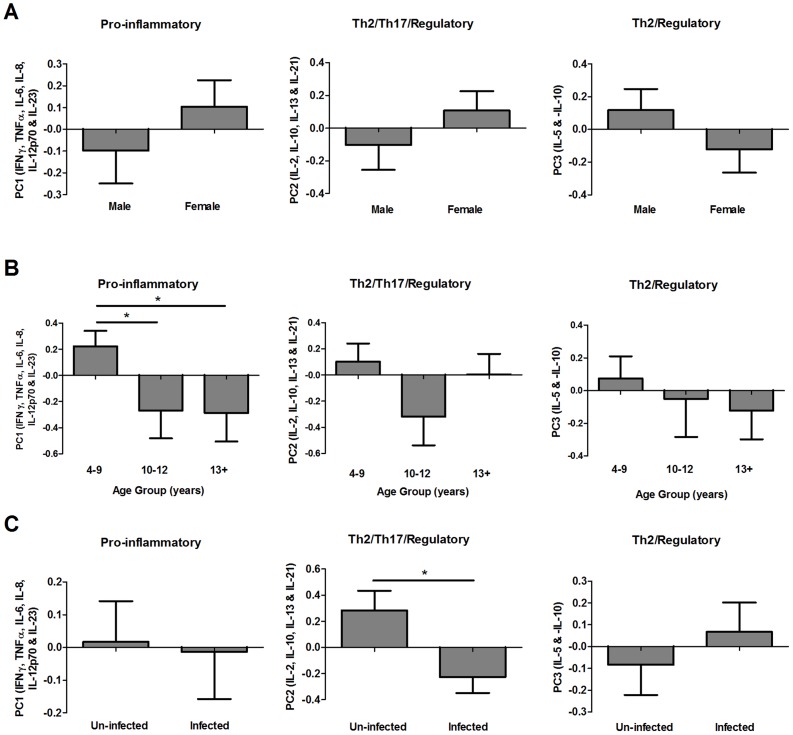
Distribution of post-treatment GST-specific cytokine profiles by gender, age group and *S. haematobium* infection status. Mean post-treatment principal component regression factor scores for cytokine profiles associated with pro-inflammatory (post-treatment PC1), Th2/Th17/regulatory (post-treatment PC2) and Th2/regulatory (post-treatment PC3) responses sub-divided by gender (**A**), age group (**B**) and schistosome infection status at baseline (**C**; un-infected = egg negative in all urine samples, infected = egg positive in one or more urine samples). Error bars: standard error of the mean. Where ANOVA (sequential sums of squares accounting for variation in gender and age group before infection status) analysis indicated that age group contributed to variation in cytokine profiles, pair-wise comparisons of PC scores were conducted via post-hoc Fisher's least significant difference tests between the 3 age groups (n = 107);*p<0.05.

Post-treatment cytokine responses produced in response to GST stimulation by >30% of participants (i.e. IFNγ, TNFα, IL-2, IL-5, IL-6, IL-8, IL-10, IL-12p70, IL-13, IL-21 and IL23p19) were reduced into post-treatment cytokine profiles via factor analysis (for factor loadings refer to Table S2). Similar to patterns observed before treatment, the majority of variation (32.5%) between post-treatment responses was due to differences in pro-inflammatory cytokine responses (IFNγ, TNFα, IL-6, IL-8, IL-12p70 and IL23p19; PC1). Variation was also evident in a combination of IL-2, IL-10, IL-13 and IL-21 levels, reflecting responses associated with Th2, regulatory and Th17 cells (PC2; 13.2% of variance), and a profile that was positively associated with the type 2 effector cytokine IL-5 and negatively associated with the regulatory cytokine IL-10 (PC3; 10.0% of variance). Post-treatment PC1 significantly differed according to participant age group (Figure 4, Table 3; F_2, 102_: 3.547, p = 0.032) with the youngest participants having significantly higher scores than those in the 10–12 (Mean difference: 0.516, p = 0.049) or 13+ age groups (Mean difference: 0.508, p = 0.026). Post-treatment PC2 scores significantly differed according to infection status at baseline and were lower in participants with patent infection at the time of treatment than in their schistosome-negative counterparts (Figure 4, Table 3; F_1, 102_: 6.070, p = 0.015), suggesting that the presence of live parasites at the time of treatment influenced levels of Th2, Th17 and regulatory type cytokines 6 weeks later. PC3 scores were not significantly affected by participant gender, age group or pre-treatment infection status (Figure 4, Table 3).

### Re-infected children have lower GST-specific IL-12p70 post-treatment

**Figure 5:**
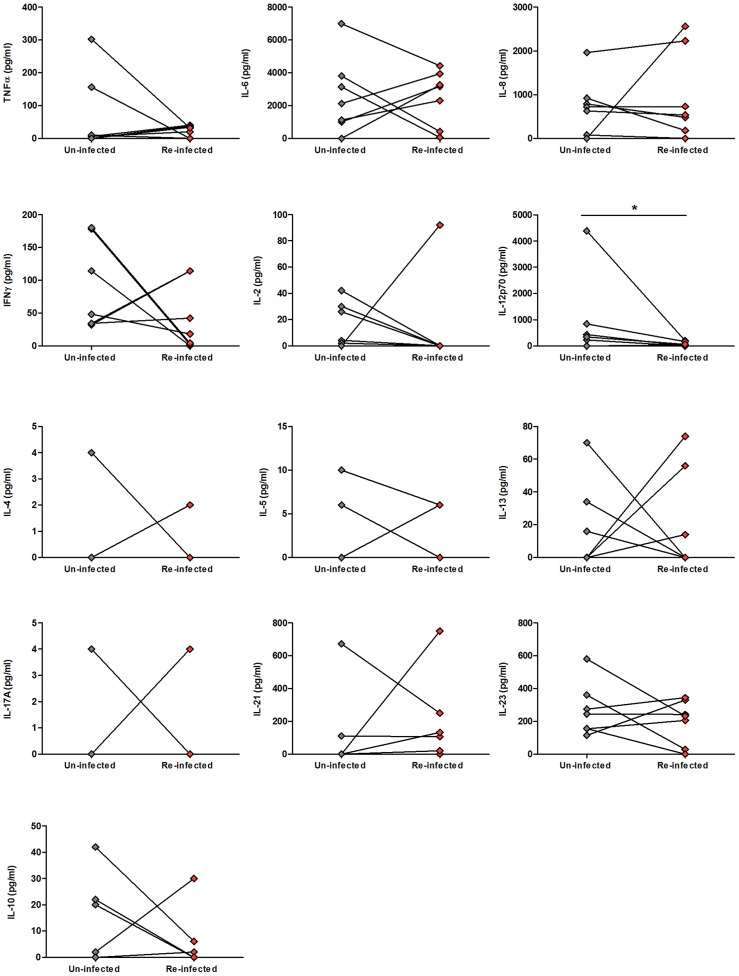
Levels of 6 weeks post-treatment GST-specific cytokines in children who remained un-infected and those who were re-infected within 18 months of treatment. The levels GST-specific (pg/ml, untransformed) cytokines associated with innate inflammatory (**top row**), Th1 (**second row**), Th2 (**third row**), Th17 (**fourth row**) and regulatory (**fifth row**) responses produced 6 weeks after treatment by un-infected children (**grey diamonds**) and age, gender and pre-treatment infection intensity-matched children who were re-infected (**red diamonds**) within 18 months of treatment (i.e. ≥1 *S. haematobium* egg in any of their urine samples collected 6 months and 18 months post-treatment). Lines join age, sex and infection intensity matched pairs and details of the characteristics of each pair are provided in **Table S3**. Results of paired Wilcoxon comparison between un-infected (n = 7) and re-infected children (n = 7) are indicated, *p<0.05.

It has been proposed that higher post-treatment schistosome-specific cytokine responses to schistosome antigens promote resistance to re-infection [10], [31] and we therefore sought to investigate whether GST-specific cytokine responses differed between participants who were re-infected within 18 months of treatment and those who remained un-infected. Only 7 participants within the cohort were re-infected within 18 months of treatment (4 males, 3 females, aged 7–13 years) and we therefore compared their 6 week post-treatment GST-specific cytokine responses to those of 7 un-infected children matched according to age, gender and pre-treatment infection status and intensity (Table S3; intensity matched by ±57.34 eggs, no other age- and gender-matched participants within the post-treatment cohort matched the pre-infection intensity of the re-infected participants). Of the 13 cytokines assayed, only post-treatment GST-specific IL-12p70 levels differed significantly between the two groups (Figure 5; Z: −1.992, p = 0.046, not significant after Bonferroni adjustment for multiple comparisons). GST-specific IL-12p70 levels were lower in the re-infected child than in the age-, gender- and pre-treatment infection intensity-matched child that remained uninfected post-treatment in 5 of the 7 pairs, higher in one pair, and the same in one pair of children (Figure 5).

## Discussion

*S. haematobium* GST has been extensively characterised as a vaccine candidate antigen for urogenital schistosomiasis in laboratory models and has also advanced further along the vaccine development pathway than any of the other potential anti-schistosome vaccines [3], [16], [30]. Despite the efficacy of GST vaccination in animal models [25], [26] and immunogenicity in clinical trials [30] the phenotype of the cellular immune response to GST in populations endemically exposed to *S. haematobium* has been largely uncharacterised. The latter is particularly the case for innate inflammatory, regulatory and Th17 associated immune markers due to the relatively recent characterisation of the role played by these immune phenotypes in human schistosomiasis [10], [49], [50]. The current study addresses this gap in our understanding of how GST-specific whole blood cytokine responses, including cytokines associated with innate inflammatory (TNFα, IL-6 and IL-8), Th1 (IFNγ, IL-2 and IL-12p70), Th2 (IL-4, IL-5 and IL-13), Th17 (IL-17A, IL-21 and IL-17A) and regulatory (IL-10) immune phenotypes, are distributed both before and after PZQ treatment in an *S. haematobium*-endemic community.

High intensity schistosome infections tend to be aggregated in school-age children and are comparatively lower in adults indicating that the relationship between infection intensity, exposure history and infection-related immune responses change with age [34]. However, the only study that has quantified cytokine responses to GST in schistosome-exposed humans focused on adults [33]. We therefore investigated a wider range of ages (4–84 years) with the hypothesis that GST-specific cytokine responses would vary between 3 age groups reflecting age-dependent changes in *S. haematobium* infection distribution; increasing intensity of infection (4–9 year olds), peaking infection intensity (10–12 year olds) and declining infection intensity (13+ year olds). Exclusion of co-infected and non-permanent residents of the study area meant that all participants included in this age stratified analysis had experienced life-long exposure to infection and thus their age was considered a proxy for their history of exposure to *S. haematobium* [34], [51].

Our results show that whole blood samples from untreated schistosome-exposed participants of all ages produce detectable cytokine responses to GST stimulation, which is consistent with observations that immune responses to GST develop naturally in both resistant and susceptible individuals [27], [33], [52] and are evident from a young age [52]. In contrast, whole blood samples from schistosome and GST naïve people do not produce detectable levels of cytokines in responses to stimulation with GST *in vitro* [30]. We also demonstrate that participant age contributes to variation in GST-specific cytokine responses in a schistosome-endemic context by affecting both the capacity to produce different types of GST-specific cytokines and patterns of co-produced cytokines. The highest percentage of GST-specific IL-4, a Th2-associated cytokine, and IL-10, a regulatory cytokine, producers was in the youngest age group (4–9 years), which may reflect the more regulatory schistosome-specific immune profile of individuals with a short history of schistosome exposure. For example, at younger ages, the proportion of circulating T regulatory cells (Treg) is more positively associated with *S. haematobium* infection intensity than in older age groups (14+ years) in whom this relationship is negatively correlated [49]. We also found that a combined phenotype of Th2 (IL-5 and IL-13) and regulatory (IL-10) cytokine responses to GST (PC2) was lowest in children aged 10–12 years in whom infection intensity and prevalence are peaking, suggesting that this may be a particularly dynamic period in terms of GST exposure and development of GST-specific cellular immune responses. Taken together these observations indicate that the Th2-type responses most associated with protective immunity in previous studies (i.e. IL-4 and IL-5 [30], [31]) are also the most age-dependent. Therefore, GST-based vaccine efficiency should be assessed across a range of ages during trials in schistosome-exposed populations in order to generate accurate predictions of population efficacy. An important area for future research will be to identify GST-specific cytokine-producing cell types within the whole blood milieu and determine whether these cells are influenced by host age and schistosome infection intensity.

Interestingly neither GST-specific whole blood cytokine production nor phenotype differed according to gender despite evidence of a gender bias in both neutralising antibody and PBMC cytokine responses identified in previous studies in adults endemically exposed to *S. haematobium* [33] and *S. mansoni* [27]. At baseline, cytokine responses also did not differ between individuals without infection and those with *S. haematobium* infection who might be expected to have stronger cytokine responses to GST due to on-going exposure to live adult worms. We also found no significant correlation between pre-treatment GST-specific cytokine profiles (PCs 1–3) and *S. haematobium* infection intensity (data not shown). The absence of an infection-related difference in GST-specific cytokines may be due to the fact that many abundant somatic antigens (including GST) are sequestered from the immune system by live schistosome worms [9],[11],[52]. Therefore, recall responses of whole blood cells to GST stimulation *in vitro* may be more closely related to a person's history of exposure to dying worms than to the presence or absence of live worms in the bloodstream [11], as described for age-related patterns of GST-specific cytokine responses above. It is also possible that exposure of un-infected individuals (i.e. egg negative urine samples) to GST-expressing schistosome larvae which do not develop into fecund adult worm pairs is sufficient to elicit similar GST-specific cytokine responses to those of individuals harbouring patent infections (i.e. egg positive urine samples) [53].

Given that PZQ treatment results in a specific increase in the intensity of GST recognition by serum antibodies [9] in addition to a generalised increase in immune responsiveness to whole adult schistosome homogenates [10], we were interested to see whether GST-specific cytokine profiles changed post-treatment. Six weeks after treatment we observed a dramatic increase in the percentage of participants producing detectable GST-specific levels of all innate inflammatory (TNFα, IL-6 and IL-8) and Th1-associated (IFNγ, IL-2 and IL-12p70) cytokines, as well as the effector Th2-associated cytokine IL-13 and the Th17-associated cytokine IL-23p19 (i.e. cytokines associated with pro-inflammatory and effector immune responses). Reflecting this boost in pro-inflammatory cytokine responses after treatment, a clear shift was evident in the combined pattern of GST-specific responses after PZQ treatment relative to baseline. The NMS analysis used to show this pattern was particularly informative since it incorporated all 13 GST-specific cytokines for each individual into a single analysis [46] and thus avoided focusing on variation in individual cytokines which are inherently cross-regulatory and therefore non-independent [54]. NMS has the added benefit of being based on relative differences in cytokine responses (i.e. each NMS score is based on levels of all 13 cytokines ranked relative to those of the rest of the cohort [46]) rather than quantitative differences between individuals and thus the analysis is not biased towards abundant cytokines which are not necessarily more bioactive than those present at low concentrations (e.g. cytokine bioactivity is dependent on cytokine receptor expression in addition to cytokine concentration [54]). Ours is the first study to show that GST-specific cytokine responses are boosted by PZQ treatment but our observations are consistent with previous observations in the same Zimbabwean community that PZQ treatment leads to an increase in pro-inflammatory cytokine responses to antigens from whole *S. haematobium* cercariae and eggs, which both express GST [10]. Both the intensity of GST recognition and the range of GST isoforms bound by serum antibodies are also enhanced in PZQ-treated inhabitants of schistosome-endemic areas relative to that observed before treatment [9], supporting our observations of a boost in cellular responsiveness specifically to GST after treatment. Increased levels of schistosome-specific cytokines have also been observed following treatment of *S. mansoni*-exposed populations, although cytokines associated with innate inflammatory and Th17 responses were not assayed in these studies [8], [13].

Following treatment, the 4–9 years age group had the highest scores for pro-inflammatory cytokine profiles relative to the older age groups. Thus, children with the least exposure to schistosome infection and the most regulatory responses to crude schistosome antigens prior to treatment [49] experience the most prominent boost in pro-inflammatory cytokine responses to GST post-treatment. A more pronounced post-treatment increase in children relative to adults has previously been observed for antibody responsiveness to schistosome antigens [55]. PZQ treatment also resulted in schistosome infection-related differences in post-treatment GST-specific Th2-/Th17-/regulatory-type cytokine responses, which were lower in participants with patent infection at the time of treatment than in their un-infected counterparts. The difference between infected and uninfected participant cytokine responses after, but not before treatment, likely reflects the pronounced increase in GST exposure resulting from a PZQ-mediated adult worm death in infected but not uninfected individuals [9], [11]. For example, the relative shift away from a Th2/Th17/regulatory profile following treatment of infected but not uninfected participants may result from a rapid decline in Th2 cells and immunosuppressive mechanisms following removal of live parasites, as described in previous studies [50], [55].

Few participants were re-infected within 18 months of treatment (n = 7), however we observed that GST-specific IL-12p70 responses were lower in these re-infected children than in their gender, age and pre-treatment infection intensity-matched counterparts who remained uninfected. Although the number of participants included in this analysis is too low to draw firm conclusions on the influence of GST-specific cytokine responses on re-infection rates, this pattern is consistent with our previous observations in the same community that lower pro-inflammatory whole blood cytokine responses to *S. haematobium* egg antigens are associated with a higher risk of re-infection [10]. Assaying GST-specific cytokine responses over a longer period after PZQ treatment or in communities experiencing more intense re-infection rates are required to identify whether GST-specific cytokine responses contribute to schistosome re-infection risk.

Collectively this study offers the first comprehensive characterisation of the distribution of GST-specific cytokine responses at a population level in humans naturally exposed to schistosome infection. Our results indicate that GST-specific cytokine profiles are influenced by participant age and PZQ treatment and post-treatment cytokine responses to GST are also influenced by pre-treatment infection intensity. Thus these factors may influence cellular responses to a GST vaccine formulation and should be taken into consideration in future immunogenicity and efficacy trials.

## Supporting Information

Figure S1 **Distribution of the percentage production and levels of individual GST-specific cytokine responses by age group.** The percentage of participants producing detectable amounts of GST-specific cytokines associated with innate inflammatory (**top row**), Th1 (**second row**), Th2 (**third row**), Th17 (**fourth row**) and regulatory (**fifth row**) type cellular immune phenotypes and the levels of these cytokines produced (pg/ml, untransformed) in the 4–9, 10–12 and 13+ age groups at baseline (n = 195). Median values are indicated by horizontal bars. Pearson's Chi-squared comparisons of percentage production between age groups and by gender and *S. haematobium* infection status are provided in **Table 2**.
(TIF)

Figure S2 **GST-specific cytokine production before and 6 weeks after praziquantel treatment.** The percentage of participants producing detectable amounts of GST-specific cytokines associated with innate inflammatory (**top row**), Th1 (**second row**), Th2 (third row), Th17 (**fourth row**) and regulatory (**fifth row**) type cellular immune phenotypes and the levels of these cytokines produced (pg/ml, untransformed) before (**grey triangles**) and 6 weeks after (**red triangles**) a single dose of praziquantel (n = 107). Median values are indicated by horizontal bars. McNemar comparisons of percentage production before and after treatment are provided in **Table 4**.
(TIF)

Table S1 **Factor analysis of GST-specific cytokine responses before anti-helminthic treatment.** ^a^Factor loadings for each PC (columns) are indicated for individual cytokines (arranged in rows according to the cellular immune phenotype with which they aremost commonly associated). Cytokines with factor loadings ≥0.5 or ≤−0.5 were considered to significantly contribute to the PC (underlined). *^b^GST-specific cytokines produced by <30% of participants were not included in the factor analysis.
(DOCX)

Table S2 **Factor analysis of GST-specific cytokine responses 6 weeks post-treatment.** ^a^Factor loadings for each PC (columns) are indicated for individual cytokines (arranged in rows according to the cellular immune phenotype with which they are most commonly associated). Cytokines with factor loadings ≥0.5 or ≤−0.5 were considered to significantly contribute to the PC (underlined). *^b^GST-specific cytokines produced by <30% of participants were not included in the factor analysis.
(DOCX)

Table S3 **Characteristics of children re-infected within 18 months of treatment and their age-, sex- and** ***S. haematobium*** **infection-matched pairs who remained un-infected post-treatment.**
(DOCX)
