# Supplementary figures and images for: Cytokine Responses to the Anti-schistosome Vaccine Candidate Antigen Glutathione-S-transferase Vary with Host Age and Are Boosted by Praziquantel Treatment
Source: PLoS Negl Trop Dis. 2014 May 8;8(5):e2846. doi: 10.1371/journal.pntd.0002846 (PMC4014416; doi:10.1371/journal.pntd.0002846)

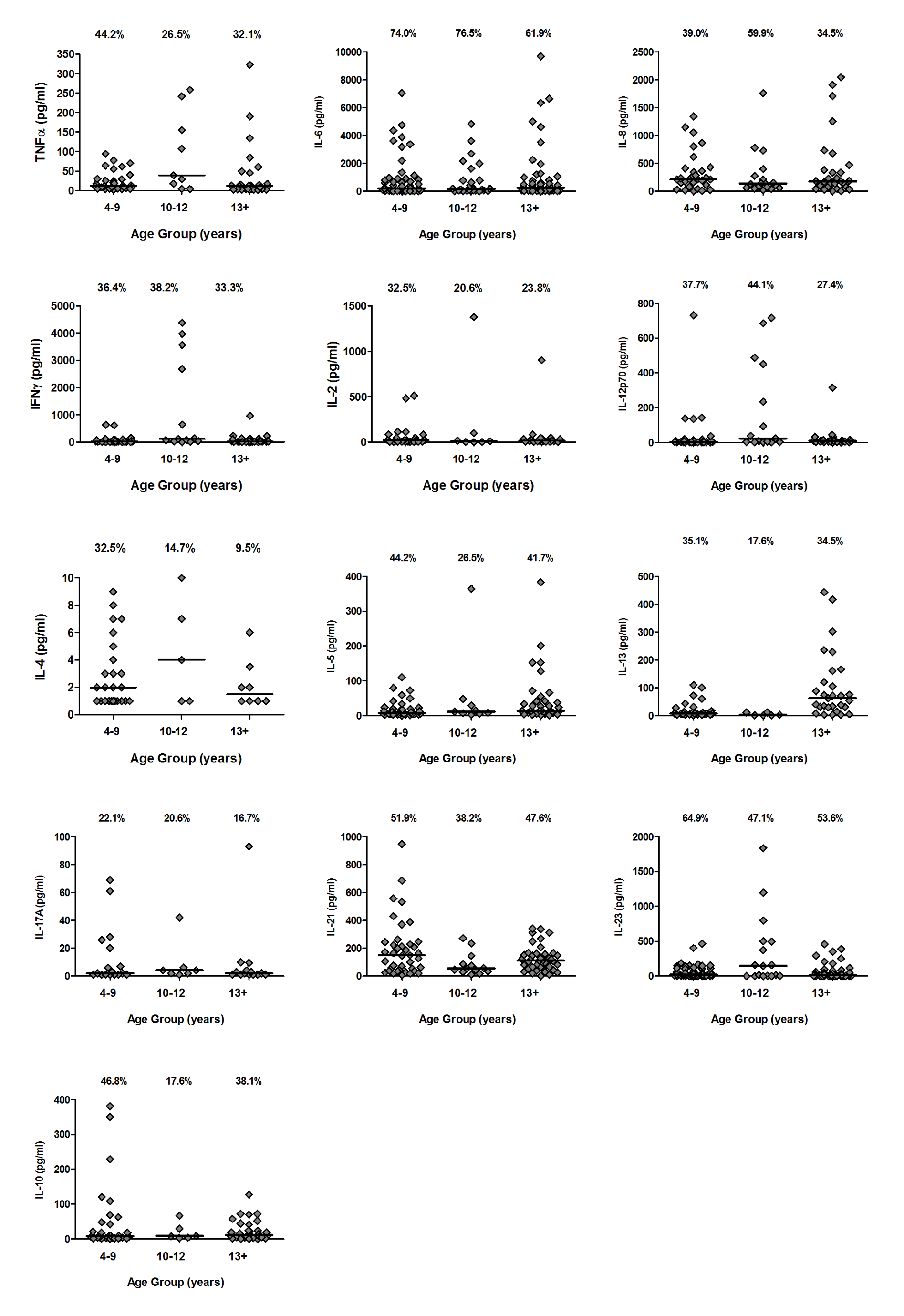

Supplement: Figure S1 — Distribution of the percentage production and levels of individual GST-specific cytokine responses by age group. The percentage of participants producing detectable amounts of GST-specific cytokines associated with innate inflammatory (top row), Th1 (second row), Th2 (third row), Th17 (fourth row) and regulatory (fifth row) type cellular immune phenotypes and the levels of these cytokines produced (pg/ml, untransformed) in the 4–9, 10–12 and 13+ age groups at baseline (n = 195). Median values are indicated by horizontal bars. Pearson's Chi-squared comparisons of percentage production between age groups and by gender and S. haematobium infection status are provided in Table 2 . (TIF) [file pntd.0002846.s001.tif]

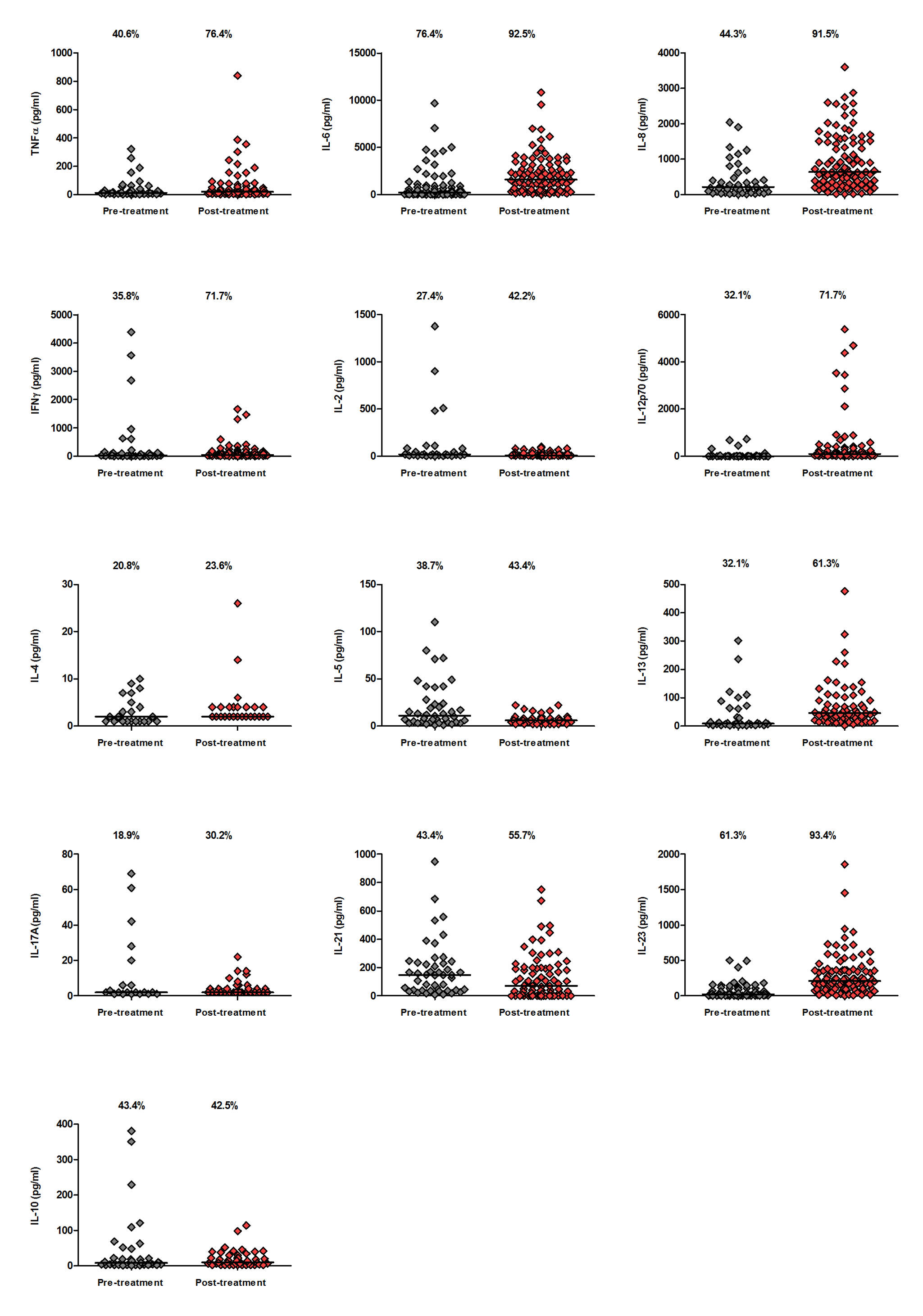

Supplement: Figure S2 — GST-specific cytokine production before and 6 weeks after praziquantel treatment. The percentage of participants producing detectable amounts of GST-specific cytokines associated with innate inflammatory (top row), Th1 (second row), Th2 (third row), Th17 (fourth row) and regulatory (fifth row) type cellular immune phenotypes and the levels of these cytokines produced (pg/ml, untransformed) before (grey triangles) and 6 weeks after (red triangles) a single dose of praziquantel (n = 107). Median values are indicated by horizontal bars. McNemar comparisons of percentage production before and after treatment are provided in Table 4 . (TIF) [file pntd.0002846.s002.tif]
