# Supplementary material for: Cytokine Responses to the Anti-schistosome Vaccine Candidate Antigen Glutathione-S-transferase Vary with Host Age and Are Boosted by Praziquantel Treatment
Source: PLoS Negl Trop Dis. 2014 May 8;8(5):e2846. doi: 10.1371/journal.pntd.0002846 (PMC4014416; doi:10.1371/journal.pntd.0002846)
Supplement: Table S1 — Factor analysis of GST-specific cytokine responses before anti-helminthic treatment. aFactor loadings for each PC (columns) are indicated for individual cytokines (arranged in rows according to the cellular immune phenotype with which they aremost commonly associated). Cytokines with factor loadings ≥0.5 or ≤−0.5 were considered to significantly contribute to the PC (underlined). *bGST-specific cytokines produced by <30% of participants were not included in the factor analysis. (DOCX) [file pntd.0002846.s003.docx]

**Table S1: Factor analysis of GST-specific cytokine responses before anti-helminthic treatment**

|  |  | **Principal Component^a^** | | |
| --- | --- | --- | --- | --- |
|  |  | **1** | **2** | **3** |
|  |  | **Pro-inflammatory** | **Th2/Regulatory** | **Innate Inflammatory/**  **Th1** |
|  | **TNFα** | **0.7** | 0.2 | 0.1 |
| **Innate Inflammatory** | **IL-6** | **0.8** | -0.1 | 0.3 |
|  | **IL-8** | **0.6** | -0.3 | **0.6** |
|  | **IFNγ** | **0.6** | -0.1 | **-0.6** |
| **Th1** | **IL-2** | *^b^ | *^b^ | *^b^ |
|  | **IL-12p70** | **0.7** | 0.1 | -0.4 |
|  | **IL-4** | *^b^ | *^b^ | *^b^ |
| **Th2** | **IL-5** | 0.1 | **0.6** | 0.1 |
|  | **IL-13** | 0.0 | **0.6** | 0.0 |
|  | **IL-17A** | *^b^ | *^b^ | *^b^ |
| **Th17** | **IL-21** | 0.2 | 0.4 | -0.2 |
|  | **IL-23** | **0.8** | -0.1 | 0.0 |
| **Regulatory** | **IL-10** | 0.1 | **0.6** | 0.2 |
| **% of variance** |  | **28.5** | **14.3** | **10.5** |

^a^Factor loadings for each PC (columns) are indicated for individual cytokines (arranged in rows according to the cellular immune phenotype with which they aremost commonly associated). Cytokines with factor loadings ≥0.5 or ≤ -0.5 were considered to significantly contribute to the PC (underlined).

*^b^GST-specific cytokines produced by <30% of participants were not included in the factor analysis
