# Supplementary material for: Cytokine Responses to the Anti-schistosome Vaccine Candidate Antigen Glutathione-S-transferase Vary with Host Age and Are Boosted by Praziquantel Treatment
Source: PLoS Negl Trop Dis. 2014 May 8;8(5):e2846. doi: 10.1371/journal.pntd.0002846 (PMC4014416; doi:10.1371/journal.pntd.0002846)
Supplement: Table S2 — Factor analysis of GST-specific cytokine responses 6 weeks post-treatment. aFactor loadings for each PC (columns) are indicated for individual cytokines (arranged in rows according to the cellular immune phenotype with which they are most commonly associated). Cytokines with factor loadings ≥0.5 or ≤−0.5 were considered to significantly contribute to the PC (underlined). *bGST-specific cytokines produced by <30% of participants were not included in the factor analysis. (DOCX) [file pntd.0002846.s004.docx]

**Table S2: Factor analysis of GST-specific cytokine responses 6 weeks post-treatment**

|  |  | **Principal Component^a^** | | |
| --- | --- | --- | --- | --- |
|  |  | **1** | **2** | **3** |
|  |  | **Pro-inflammatory** | **Th2/Th17Regulatory** | **Th2/Regulatory** |
|  | **TNFα** | **0.7** | -0.1 | -0.1 |
| **Innate Inflammatory** | **IL-6** | **0.9** | -0.2 | 0.0 |
|  | **IL-8** | **0.6** | 0.1 | 0.0 |
|  | **IFNγ** | **0.7** | -0.1 | -0.3 |
| **Th1** | **IL-2** | 0.0 | **0.6** | 0.2 |
|  | **IL-12p70** | **0.7** | -0.2 | 0.2 |
|  | **IL-4** | *^b^ | *^b^ | *^b^ |
| **Th2** | **IL-5** | 0.3 | 0.3 | **0.7** |
|  | **IL-13** | 0.3 | **0.5** | 0.3 |
|  | **IL-17A** | *^b^ | *^b^ | *^b^ |
| **Th17** | **IL-21** | 0.2 | **0.6** | -0.3 |
|  | **IL-23** | **0.9** | -0.1 | -0.1 |
| **Regulatory** | **IL-10** | 0.1 | **0.5** | **-0.6** |
| **% of variance** |  | **32.5** | **13.2** | **10.0** |

^a^Factor loadings for each PC (columns) are indicated for individual cytokines (arranged in rows according to the cellular immune phenotype with which they are most commonly associated). Cytokines with factor loadings ≥0.5 or ≤ -0.5 were considered to significantly contribute to the PC (underlined).

*^b^GST-specific cytokines produced by <30% of participants were not included in the factor analysis
