# Supplementary material for: Cytokine Responses to the Anti-schistosome Vaccine Candidate Antigen Glutathione-S-transferase Vary with Host Age and Are Boosted by Praziquantel Treatment
Source: PLoS Negl Trop Dis. 2014 May 8;8(5):e2846. doi: 10.1371/journal.pntd.0002846 (PMC4014416; doi:10.1371/journal.pntd.0002846)
Supplement: Table S3 — Characteristics of children re-infected within 18 months of treatment and their age-, sex- and S. haematobium infection-matched pairs who remained un-infected post-treatment. (DOCX) [file pntd.0002846.s005.docx]

**Table S3: Characteristics of children re-infected within 18 months of treatment and their age-, sex- and *S. haematobium* infection-matched pairs who remained un-infected post-treatment**

| **Pair#** | **Age**  **(years)** | **Gender** | **Baseline infection status** | **Baseline infection intensity** | |  | **Post-treatment infection intensity** | |
| --- | --- | --- | --- | --- | --- | --- | --- | --- |
|  |  |  |  | **Un-infected** | **Re-infected** |  | **Un-infected** | **Re-infected** |
| **1** | 7 | Female | Infected | 2.67 | 6.00 |  | 0.00 | 0.33 |
| **2** | 7 | Female | Infected | 12.33 | 10.67 |  | 0.00 | 1.67 |
| **3** | 11 | Female | Un-infected | 0.00 | 0.00 |  | 0.00 | 0.33 |
| **4** | 8 | Male | Infected | 1.00 | 2.67 |  | 0.00 | 104.33 |
| **5** | 9 | Male | Infected | 80.67 | 23.33 |  | 0.00 | 2.50 |
| **6** | 12 | Male | Infected | 19.33 | 23.67 |  | 0.00 | 2.67 |
| **7** | 13 | Male | Infected | 25.33 | 23.00 |  | 0.00 | 2.67 |
